# Supplementary material for: Vitamin D Modified DSS-Induced Colitis in Mice via STING Signaling Pathway
Source: Biology (Basel). 2025 Jun 18;14(6):715. doi: 10.3390/biology14060715 (PMC12190092; doi:10.3390/biology14060715)
Supplement: Supplementary file 1 [file biology-14-00715-s001.zip › Supplementary Materials S1: 16s-report of gut microbiota/Groups/Graphlan_Analysis/Group/graphlan_group.pdf]

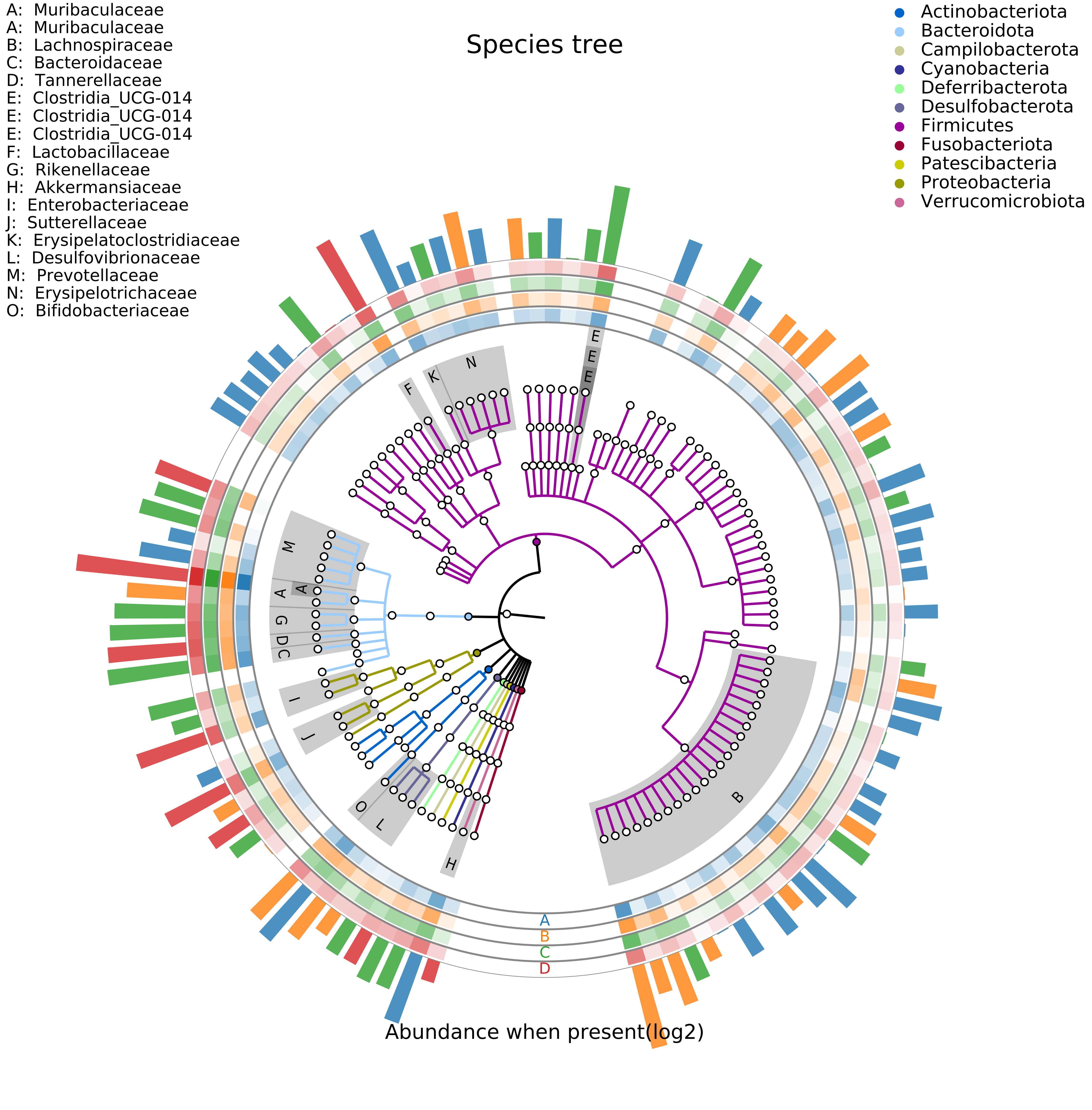

Species tree
A: Muribaculaceae
A: Muribaculaceae
B: Lachnospiraceae
C: Bacteroidaceae
D: Tannerellaceae
E: Clostridia_UCG-014
E: Clostridia_UCG-014
E: Clostridia_UCG-014
F: Lactobacillaceae
G: Rikenellaceae
H: Akkermansiaceae
I: Enterobacteriaceae
J: Sutterellaceae
K: Erysipelatoclostridiaceae
L: Desulfovibrionaceae
M: Prevotellaceae
N: Erysipelotrichaceae
O: Bifidobacteriaceae
Actinobacteriota
Bacteroidota
Campilobacterota
Cyanobacteria
Deferribacterota
Desulfobacterota
Firmicutes
Fusobacteriota
Patescibacteria
Proteobacteria
Verrucomicrobiota
A
B
C
D
Abundance when present(log2)
